# Network-based integrated analysis for toxic effects of high-concentration formaldehyde inhalation exposure through the toxicogenomic approach

**DOI:** 10.1038/s41598-022-09673-0

**Published:** 2022-04-04

**Authors:** Doo Seok Kang, Nahyun Lee, Dong Yeop Shin, Yu Jin Jang, Su-Hyon Lee, Kyung-Min Lim, Yeon-Soon Ahn, Cheol Min Lee, Young Rok Seo

**Affiliations:** 1 Department of Life Science, Dongguk University Biomedi Campus, Goyang, Gyeonggi 10326 Republic of Korea; 2 College of Pharmacy, Ewha Womans University, Seoul, 03760 Republic of Korea; 3 R&D Institute, Biosolution Co., Ltd., Seoul, 01811 Republic of Korea; 4 Department of Preventive Medicine and Institute of Occupational and Environmental Medicine, Wonju College of Medicine, Yonsei University, Wonju, Gangwon 26426 Republic of Korea; 5 Department of Chemical and Biological Engineering, College of Natural Science and Engineering, Seokyeong University, Seoul, 02173 Republic of Korea

**Keywords:** Biomarkers, Health care

## Abstract

Formaldehyde is a colorless, pungent, highly reactive, and toxic environmental pollutant used in various industries and products. Inhaled formaldehyde is a human and animal carcinogen that causes genotoxicity, such as reactive oxygen species formation and DNA damage. This study aimed to identify the toxic effects of inhaled formaldehyde through an integrated toxicogenomic approach utilizing database information. Microarray datasets (GSE7002 and GSE23179) were collected from the Gene Expression Omnibus database, and differentially expressed genes were identified. The network analyses led to the construction of the respiratory system-related biological network associated with formaldehyde exposure, and six upregulated hub genes (*AREG*, *CXCL2*, *HMOX1*, *PLAUR*, *PTGS2*, and *TIMP1*) were identified. The expression levels of these genes were verified via qRT-PCR in 3D reconstructed human airway tissues exposed to aerosolized formaldehyde. Furthermore, *NRARP* was newly found as a potential gene associated with the respiratory and carcinogenic effects of formaldehyde by comparison with human in vivo and in vitro formaldehyde-exposure data. This study improves the understanding of the toxic mechanism of formaldehyde and suggests a more applicable analytic pipeline for predicting the toxic effects of inhaled toxicants.

## Introduction

Formaldehyde is a toxic chemical with a colorless, pungent odor, and high reactivity^1^. The common toxic effects of formaldehyde include irritation and damage to contacted tissues^2^. Formaldehyde induces various levels of genotoxic effects, such as *N*^2^-hydroxymethyl-dG adducts, DNA–protein crosslinks, and chromosomal damage^3,4^. The International Agency for Research on Cancer (IARC) has classified formaldehyde as a human carcinogen that causes nasopharyngeal cancer and leukemia^1^. Despite its toxic effects, formaldehyde is ubiquitous in the environment because of its wide use in resin production, as a chemical intermediate, in disinfectants, medical fields, and preservatives, among other commercial purpose^2^. Humans are exposed to very low concentrations of environmental formaldehyde in daily life, primarily through inhalation^5^. Unlike the exposure in daily life, occupational exposure occurs at a wide range of concentrations, as much as 20.94 mg/m^3^ in an anatomical laboratory and 60.77 ppm in a factory, in extreme cases^2,6,7^. Therefore, people working in high-risk occupational environments are likely to be exposed to high concentrations for an extended time^8^. In addition, if a formaldehyde spill/leakage accident occurs in these occupational environments, human exposure to unusually very high concentrations of formaldehyde in the air causes external symptoms and underlying genetic modifications.

High-throughput technologies, such as microarray and RNA-sequencing, allow the analysis of large amounts of gene expression data. Gene Expression Omnibus (GEO), established by the National Center for Biotechnology Information (NCBI), is the largest public database that archives and distributes several types of high-throughput genomic data^9^. A comprehensive analysis of existing experimental data using bioinformatic tools can improve the understanding of biological phenomena. Various network strategies can be applied to information gathered from numerous independent studies for performing bioinformatic analysis to elucidate complex biological interactions^10,11^.

**Figure 1:**
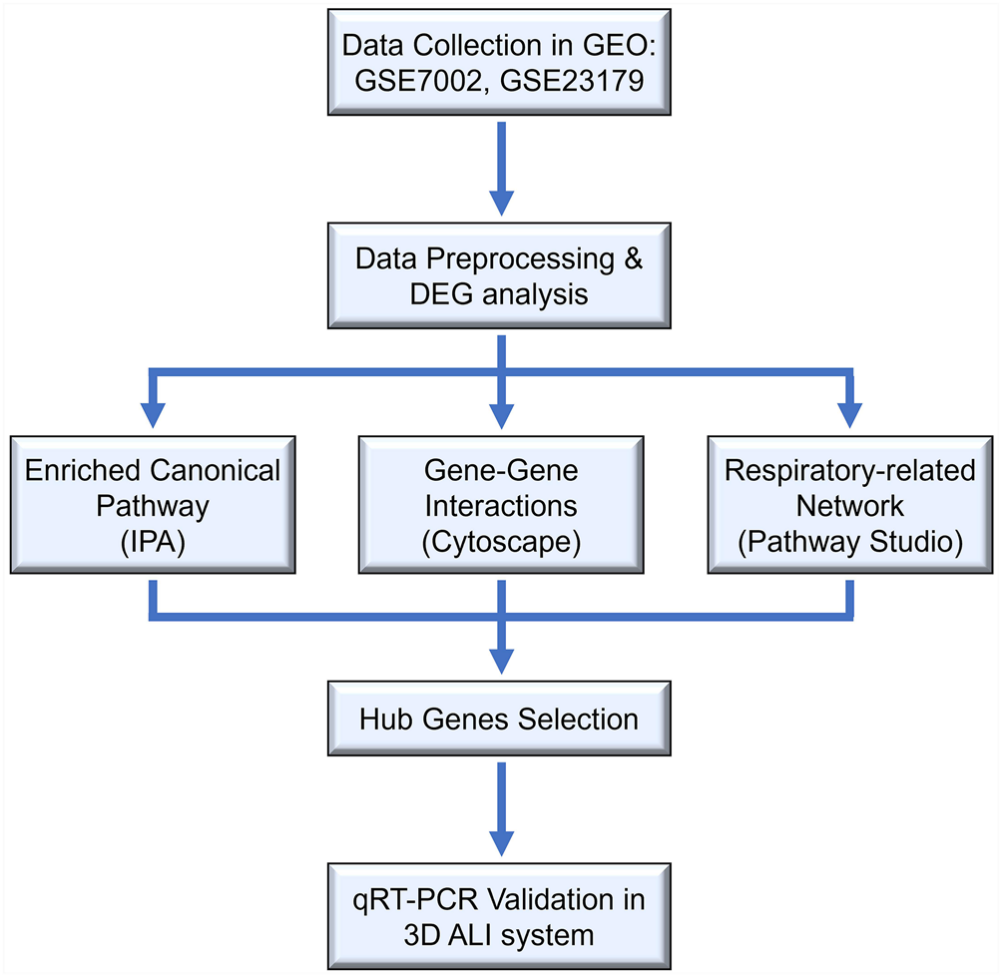
Flow chart of the steps used in this study.

To synthetically explore the toxic mechanisms of formaldehyde inhalation, two microarray datasets (GSE7002 and GSE23179) derived from formaldehyde inhalation studies in rats under identical in vivo experimental conditions were collected from the GEO database. By integrating and expanding the interpretation of the original datasets^12,13^, we investigated the toxic effects of formaldehyde exposure on the respiratory system by the toxicogenomic approach. After data preprocessing, differentially expressed genes (DEGs) were obtained, and hub genes associated with the respiratory effect of formaldehyde exposure were identified through the network-based bioinformatics approaches. To resemble airway in vivo exposure, we employed a three-dimensional (3D) reconstructed human airway mucosa model using a custom-made aerosol exposure system. The cytotoxicity of aerosolized formaldehyde was estimated, and the expression levels of selected hub genes were validated via air–liquid interface (ALI) culture. Our integrated analysis could improve understanding of the toxic effects of formaldehyde exposure on the respiratory system. An overview of the methodology applied in this study is shown in Fig. 1.

## Materials and methods

### Data collection and preprocessing

Formaldehyde inhalation gene expression profile datasets were obtained from the GEO database (https://www.ncbi.nlm.nih.gov/geo/). Two gene expression profiles (GSE7002 and GSE23179) derived from the GPL1355 platform [Rat230_2] Affymetrix Rat Genome 230 2.0 Array were downloaded. Among all datasets, samples with the high-exposure concentration (15 ppm) and different exposure periods (6 h, 5 days, 4 weeks, and 13 weeks) were selected to explore the pattern of time-dependent changes induced by formaldehyde. The selected dataset consisted of 37 control samples and 16 formaldehyde-exposed samples. The raw data were normalized using the robust multi-array average method in the affy package (version 1.62.0) in R software^14^. To remove non-biological batch effects generated in the data merging process, the ComBat function in the sva package (version 3.38.0) was used^15^.

### Clustering analysis and DEGs screening

After preprocessing, Principal component analysis (PCA) was conducted to examine the pattern of gene expression profiles derived from different datasets. DEGs between control samples and formaldehyde-exposed samples were filtered using the limma R package (version 3.40.6)^16^. The threshold criteria were |fold change|> 1.5 and false discovery rate adjusted *p*-value < 0.05. The expression patterns of the common DEGs in all exposure groups were visualized through the hierarchical clustering heatmap.

### Functional enrichment pathway analysis

The enrichment pathways for the common DEGs were analyzed using the Ingenuity Pathway Analysis (IPA) software (Qiagen, Germany). The IPA is based on the Ingenuity Knowledge Base, a large, curated biomedical database. The core analysis of IPA provides diverse biological information, including canonical pathways, upstream regulators, diseases, biofunctions, and sub-networks. Statistical significance is calculated by Fisher's exact test coupled with the *z*-score algorithm^17^.

### Network analysis and hub gene selection

Pathway Studio version 12.3, a commercial text mining-based biological network analysis software, was used to identify hub genes associated with the toxic effects of formaldehyde inhalation exposure^18^. Pathway Studio enables researchers to explore biological interactions extracted from a vast number of studies and visualize these interactions through the entity and connectivity of each relation. In Pathway Studio, "entity" signifies a node, such as a gene, disease, and cell process, and "relation" signifies a biological interaction between two entities. Each relation is expressed as a connectivity (edge) on the network and supported by the number of reference studies^19^. "Direct Interactions," "Expand Pathway," and "Shortest Path" algorithms were applied to construct the functional network of the common DEGs. Topological parameters between gene–gene interactions were calculated using the NetworkAnalyzer in Cytoscape software (version 3.7.2)^20^.

### 3D reconstructed human airway mucosa model and aerosolized formaldehyde exposure

A 3D reconstructed human airway mucosa model, SoluAirway, and its media were purchased from Biosolution Co., Ltd. (Seoul, Korea). SoluAirway was placed in a 6-well plate filled with culture media (0.9 mL/well) and pre-incubated at 37 ℃ and 5% CO_2_ overnight. Formaldehyde (16%, methanol-free, ultrapure) was purchased from Polysciences, Inc. (Warrington, PA, USA) and diluted in distilled water. According to a previous study's protocol^21^, aerosol exposure to SoluAirway was conducted for 2 min using a medical nebulizer NE-U150 (OMRON Healthcare, Japan) in the experimentally designed chamber to treat 100 μL of formaldehyde and distilled water (control). Then, the tissues were incubated for 24 h. After the aerosol exposure, the apical surfaces of the tissues were washed four times with PBS (400 μL). Tissue viability was measured by the 3-(4,5-dimethylthiazol-2-yl)-2,5 diphenyltetrazolium bromide (MTT) assay. The tissues were transferred to 24-well plates filled with MTT solution (1.0 mg/mL, 200 μL), and the tissues were filled with MTT solution (100 μL). After incubation at 37 °C and 5% CO_2_ for 3 h, MTT solution was eliminated, and the tissues were submerged in isopropanol (2.0 mL) for 3 h, protected from light. Extracted formazan was transferred to a 96-well plate (200 μL/well), and absorbance was measured at 570-nm wavelength.

### Quantitative real-time reverse transcription PCR (qRT-PCR)

The membranes of SoluAirway were cut with a surgical blade, placed on a 6-cm plate, soaked in PBS, and the tissues were harvested using scrapers. Total RNA was extracted using an RNeasy kit (Qiagen, Germany), and the RNA quality was evaluated using the Agilent 2100 Bioanalyzer (Agilent Technologies, USA). cDNA was synthesized from extracted RNA (500 μg) using the ImProm-II Reverse Transcription System (Promega, USA), according to the manufacturer's instructions. qRT-PCR was conducted using TB Green Premix Ex Taq (Takara Bio, Japan) in Rotor-Gene Q (Qiagen, Germany) under the following thermal cycling conditions: initial denaturation at 95 °C for 5 min, 40 cycles of 3 steps, including 95 °C for 15 s, 60 °C for 15 s, and 72 °C for 30 s. Glyceraldehyde-3-phosphate dehydrogenase (*GAPDH*) was used to normalize the gene expression levels according to the 2^−ΔΔ*CT*^ method^22^. The sequences of primers for qRT-PCR are described in Supplementary Table S1.

### Statistical analysis

Statistical analyses were performed using R software (version 3.6.3). All experimental data are presented as the mean ± standard error of the mean (SEM). Significant differences between the formaldehyde exposure group and control were estimated by Student's *t*-test at *p*-value < 0.05.

## Results

### Clustering patterns of transcriptomic profiles and DEGs identification

**Figure 2:**
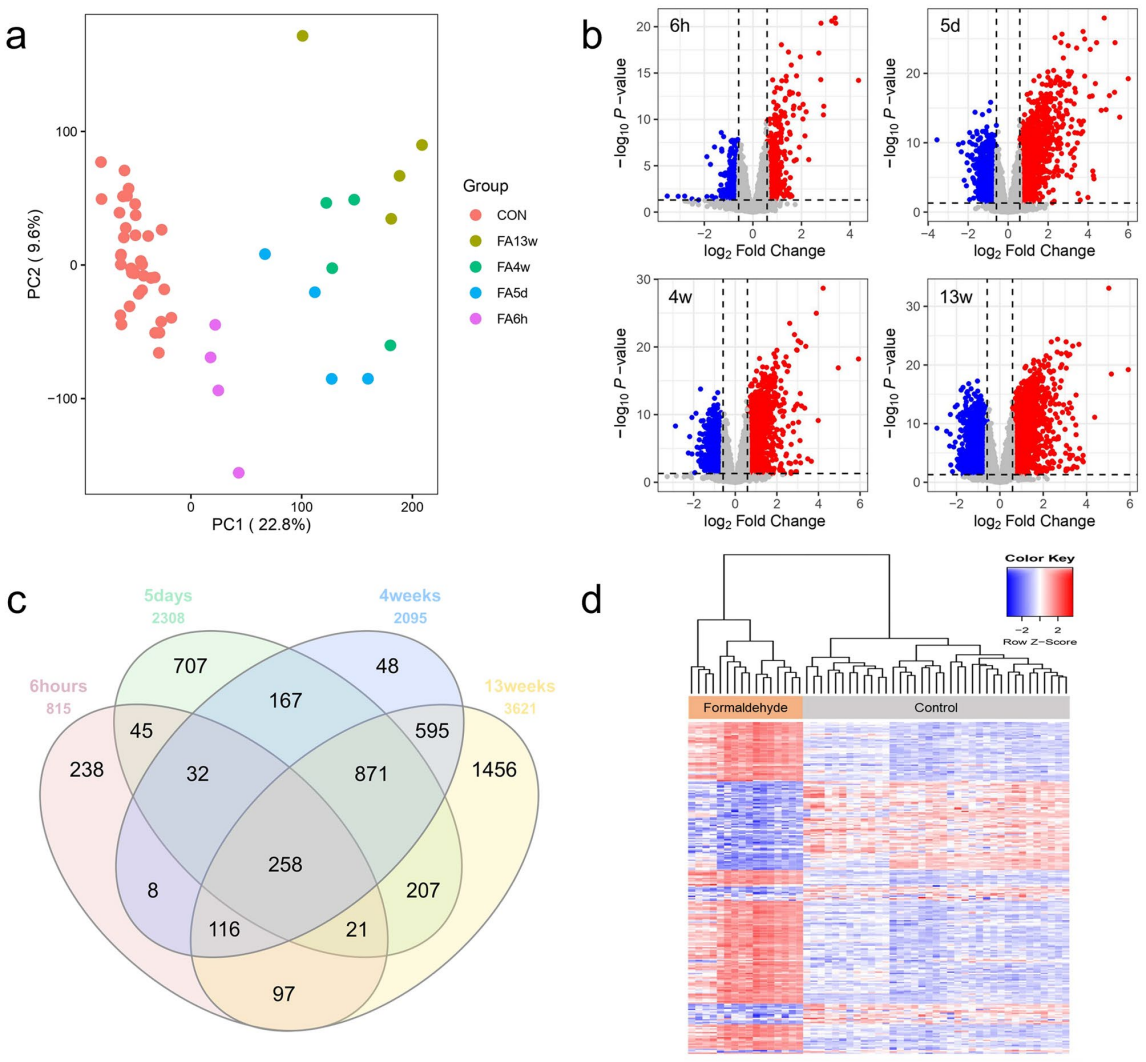
Clustering patterns of transcriptomic profiles and DEGs identification (**a**) Batch effect corrected-PCA result. PCA showed a distinct difference between the control and the exposure groups. (**b**) Volcano plot results of DEGs by each exposure groups. (**c**) Venn diagram of the DEGs among the formaldehyde exposure groups. (**d**) Hierarchical clustering heatmap of the common DEGs. All analyses were performed using R software (version 3.6.3).

The experimental conditions of the two GEO datasets (GSE7002 and GSE23179) were designed almost identically: 8-week-old male rats, whole-body inhalation exposure, 6 h/day of formaldehyde exposure, nasal epithelial cells. To acquire more robust gene expression profiles, we merged 37 control and 16 formaldehyde-exposed samples in the two GEO datasets and corrected batch effects arising from different sampling dates. PCA result indicated that formaldehyde-exposed samples were distinct from control samples, regardless of the GEO datasets (Fig. 2a).

DEGs were identified for each formaldehyde exposure group compared to the control group (Fig. 2b). There were 815 genes significantly expressed at 6 h exposure (upregulated: 354, downregulated: 461), 2,308 genes at 5 days (upregulated: 1,342, downregulated: 966), 2,095 genes at 4 weeks (upregulated: 1,118, downregulated: 977), and 3,621 genes at 13 weeks (upregulated: 1,712, downregulated: 1,909). Among all exposure groups, 258 genes were identified as common DEGs (Fig. 2c). These common DEGs showed a mostly consistent up/downregulated expression pattern at each exposure period, except for 7 genes (Fig. 2d).

### Enriched canonical pathways associated with formaldehyde exposure

**Figure 3:**
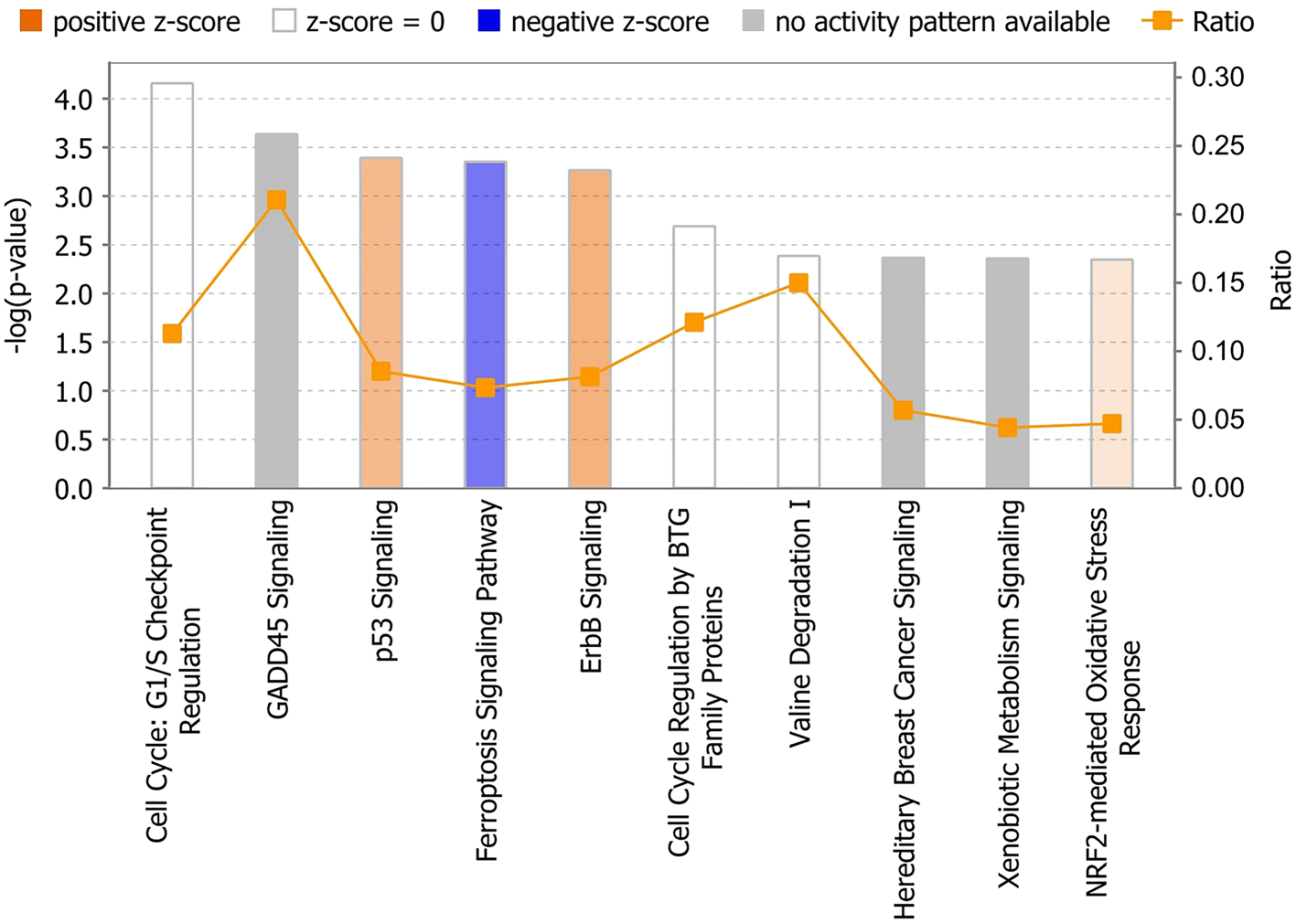
IPA top 10 canonical pathways for the common DEGs. Canonical pathways were sorted by − log(*p*-value). A positive *z*-score (orange) denotes pathway activation, and a negative *z*-score (blue) denotes pathway inhibition. Ratio refers to the percentage of DEGs among the total number of genes that make up the pathway.

To interpret the meaning of the observed gene expression changes, IPA was used to identify canonical pathways for the common DEGs filtered by human genes. The top 10 canonical pathways were selected based on the − log(*p*-value), and the activation *z*-score pattern of the pathways was considered (Fig. 3, Table 1). Among the canonical pathways, p53 Signaling (*z*-score = 1, *p* = 4.07 × 10^–4^), ErbB Signaling (*z*-score = 1.13, *p* = 5.37 × 10^–4^), and NRF2-mediated Oxidative Stress Response (*z*-score = 0.378, *p* = 4.47 × 10^–3^) exhibited positive activity patterns, and Ferroptosis Signaling Pathway (*z*-score = − 1.41, *p* = 4.47 × 10^–4^) exhibited negative activation. No activity patterns were observed in other canonical pathways.

**Table 1:** List of top 10 canonical pathways for the common DEGs.

Ingenuity canonical pathways	− log(*p*-value)	Ratio	*z*-score	Molecules
Cell Cycle: G1/S Checkpoint Regulation	4.16	0.113	0	*BTRC, CCNE2, CDKN1A, GNL3, MYC, NRG1, PAK1IP1*
GADD45 Signaling	3.64	0.211	NULL	*CCNE2, CDKN1A, GADD45B, PCNA*
p53 Signaling	3.39	0.0854	1	*CDKN1A, COQ8A, GADD45B, GNL3, PCNA, PIK3C2G, SFN*
Ferroptosis Signaling Pathway	3.35	0.0734	− 1.414	*CDKN1A, GCH1, H2AX, HMOX1, HSPB1, PRKAG2, SLC1A5, SLC7A11*
ErbB Signaling	3.27	0.0814	1.134	*AREG, EREG, HBEGF, MAP2K3, MAP2K6, NRG1, PIK3C2G*
Cell Cycle Regulation by BTG Family Proteins	2.69	0.121	NULL	*CCNE2, NOCT, PPM1L, PPP2R2B*
Valine Degradation I	2.39	0.15	NULL	*ACADSB, BCAT2, BCKDHB*
Hereditary Breast Cancer Signaling	2.37	0.0569	NULL	*CDKN1A, GADD45B, H2AX, NPM1, PIK3C2G, SFN, SMARCA2*
Xenobiotic Metabolism Signaling	2.36	0.0442	NULL	*FMO1, FMO2, GSTM2, HMOX1, MAP2K3, MAP2K6, MAP3K8, PIK3C2G, PPM1L, PPP2R2B*
NRF2-mediated Oxidative Stress Response	2.35	0.0471	0.378	*CBR1, FMO1, FOSL1, GSTM2, HMOX1, MAFF, MAP2K3, MAP2K6, PIK3C2G*

*DEG* differentially expressed gene.

### Respiratory-related network associated with formaldehyde exposure

**Figure 4:**
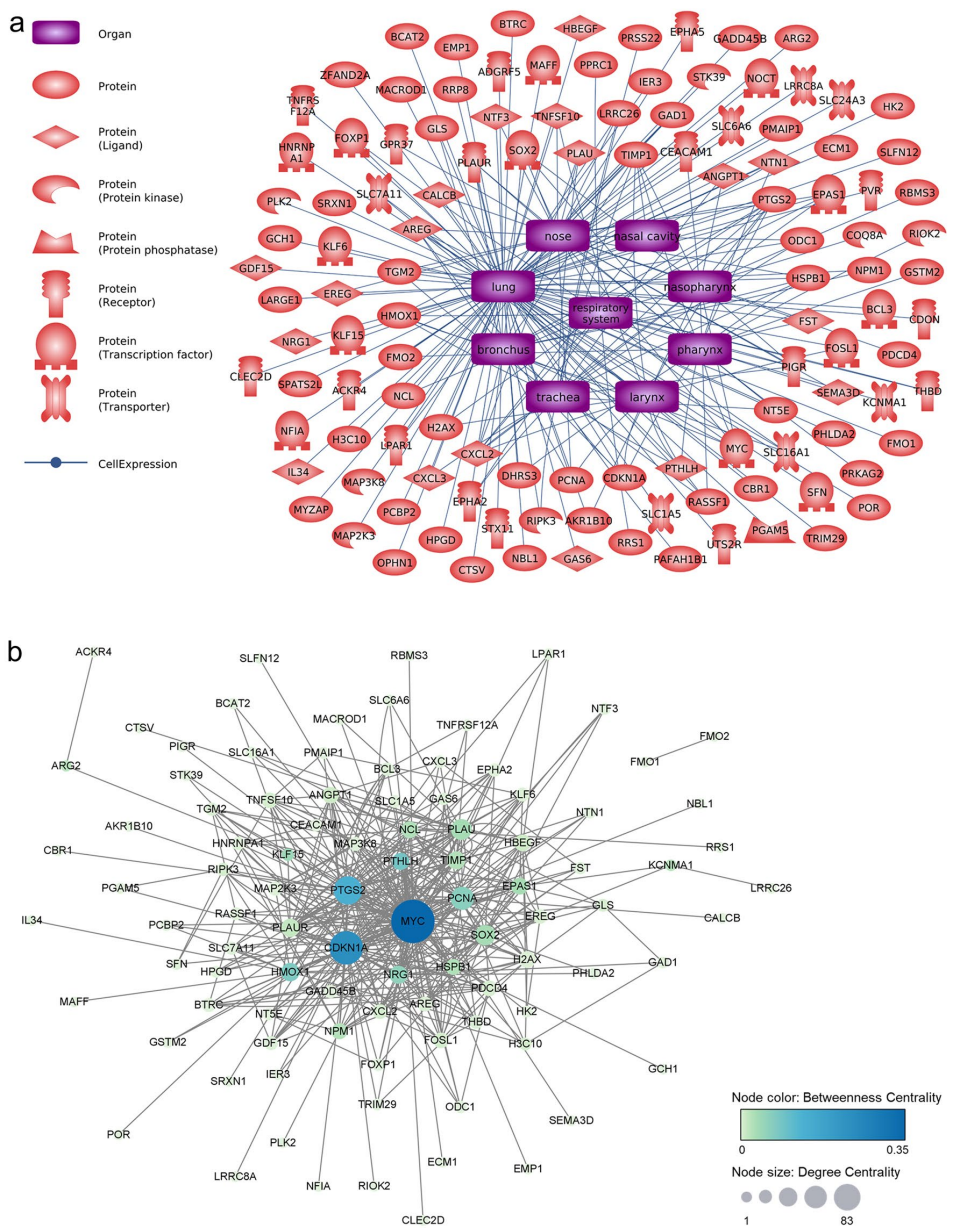

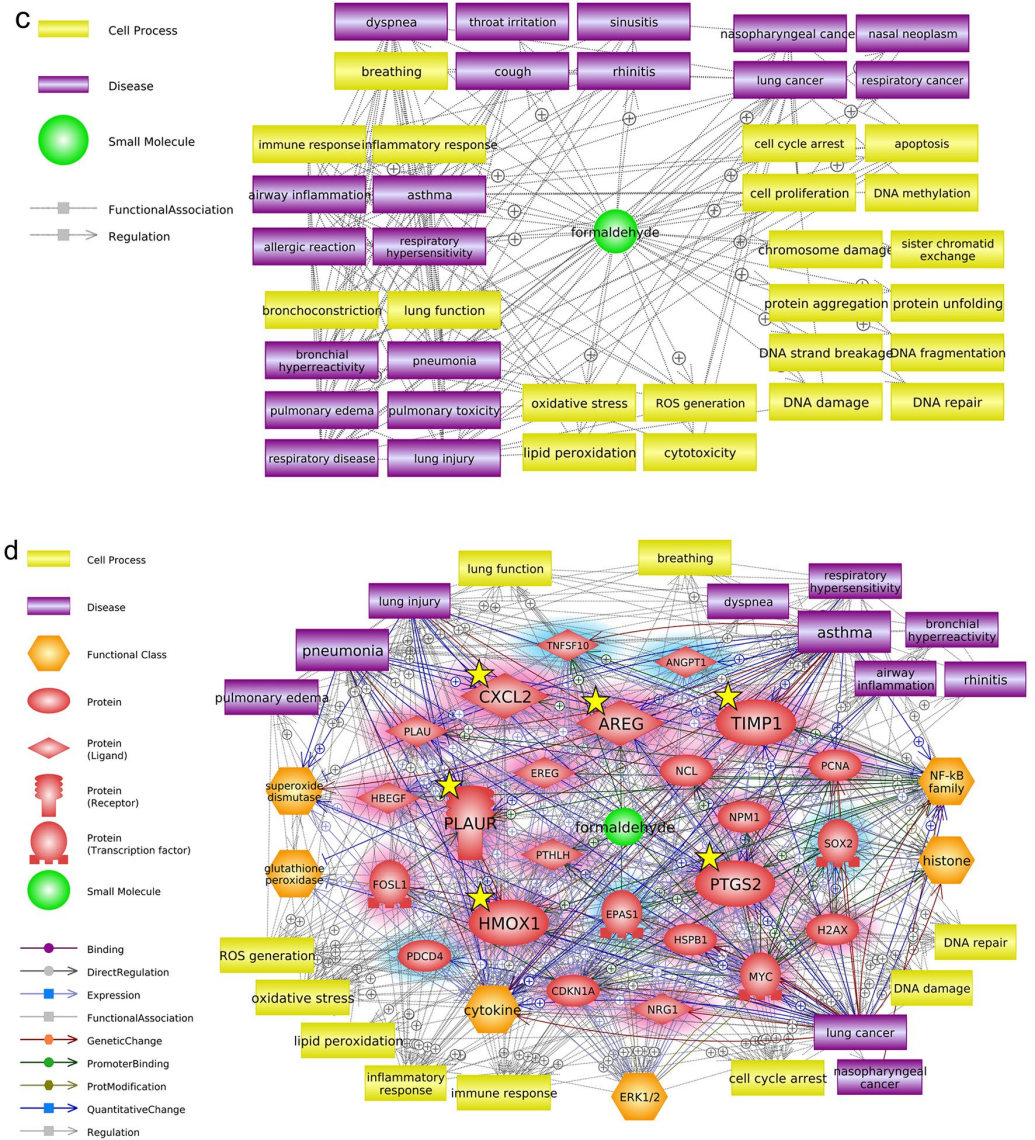
Biological networks for respiratory effects of formaldehyde exposure. (**a**) Genes with respiratory system expression information. Among the common DEGs, only genes with expression information in major respiratory organs were selected using Pathway Studio (version 12.3). (**b**) Gene–gene interaction network. Topological parameters among the genes were calculated using Cytoscape (version 3.7.2). (**c**) Formaldehyde-related major cell processes and respiratory diseases. (**d**) Respiratory system-related biological network associated with formaldehyde exposure. The biological network was constructed based on the results of the previous network analysis. Red and blue highlights, respectively, indicate up- and downregulated DEGs.

**Figure 5:**
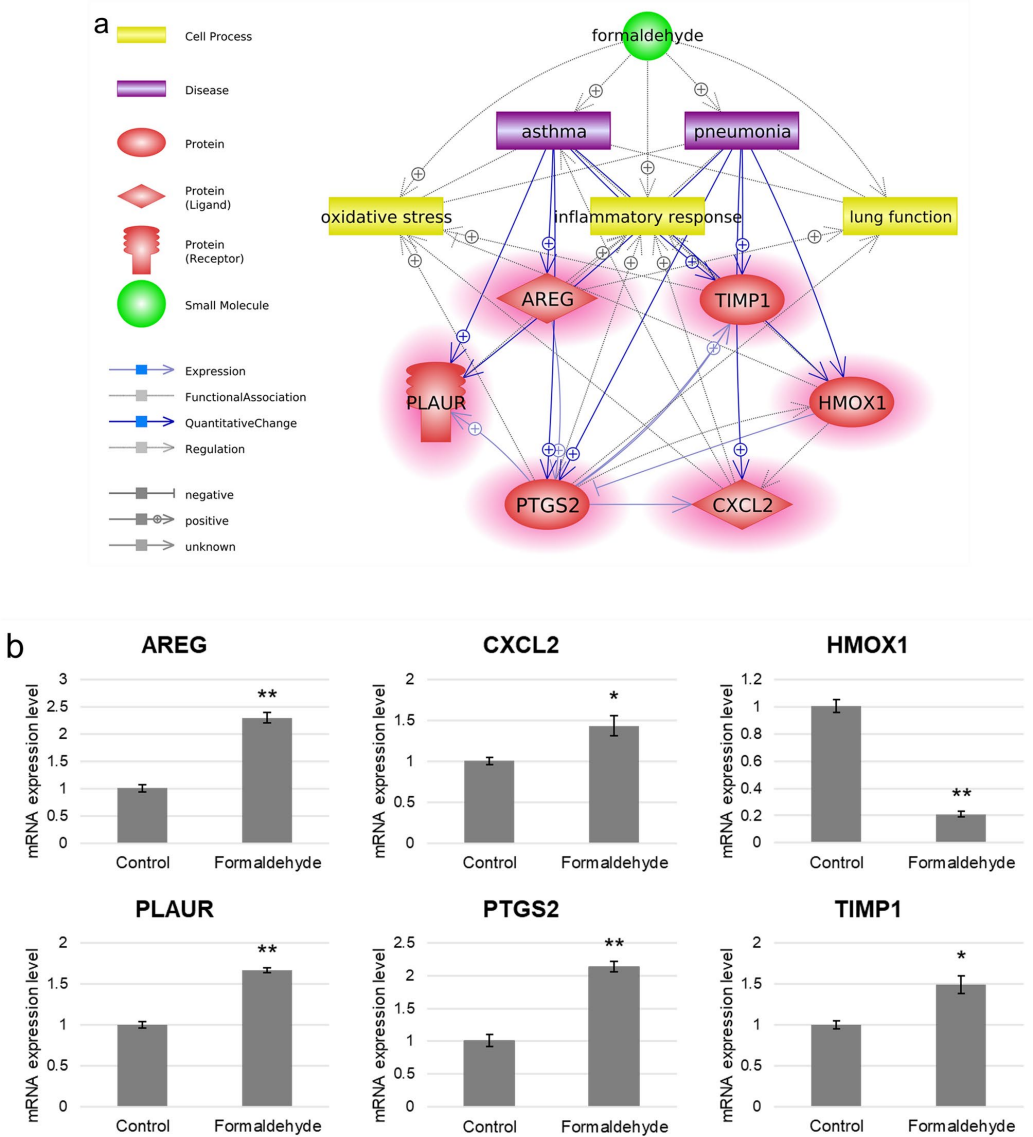
Validation of hub genes in SoluAirway. (**a**) Summarized core interactions of hub genes using Pathway Studio (version 12.3). Red highlights indicate upregulated DEGs. (**b**) Validation of the expression levels of hub genes using qRT-PCR. Error bars represent ± SEM. Single asterisk (*) and double asterisks (**) indicate *p*-value < 0.05 and < 0.01, respectively.

To identify the hub genes related to formaldehyde exposure, we used Pathway Studio to perform network analysis on the common DEGs that were most sensitive to formaldehyde exposure. We focused our analysis on the respiratory effects of inhaled formaldehyde exposure, so we filtered the genes with expression information in the major respiratory organs (from nose to lung) among the common DEGs (Fig. 4a). From these genes, we selected the top 24 genes with edge degree number ≥ 10 based on the betweenness centrality of gene–gene interactions derived from the Pathway Studio database information (Fig. 4b, Table 2). In addition, we extracted major cell processes and respiratory diseases associated with formaldehyde exposure based on the minimum number of references (≥ 3) (Fig. 4c). A biological network for respiratory effects of formaldehyde exposure was constructed with comprehensive consideration of the results of gene expression values, the centrality of gene–gene interactions, and chemical–gene–disease associations (Fig. 4d). These genes were highly associated with diseases, including lung cancer, asthma, and pneumonia, and with oxidative stress, inflammatory response, and immune response, as cell processes. Finally, six hub genes (*AREG*, *CXCL2*, *HMOX1*, *PLAUR*, *PTGS2*, and *TIMP1*) and major entities were selected through careful examination of reference studies (Fig. 5a). Moreover, functional changes related to formaldehyde exposure were predicted; activation of cell signaling (NF-κB, ERK1/2), cytokine release, histone crosslinks, and degradation of antioxidant functions (superoxide dismutase, glutathione peroxidase).

**Table 2:** Centrality information of selected genes.

Gene name	Betweenness centrality	Degree centrality	Gene name	Betweenness centrality	Degree centrality
*MYC*	0.3520	83	*TIMP1*	0.0259	19
*CDKN1A*	0.2357	58	*NPM1*	0.0247	14
*PTGS2*	0.1379	45	*PLAUR*	0.0137	20
*PTHLH*	0.0874	17	*ANGPT1*	0.0092	15
*HMOX1*	0.0773	20	*HBEGF*	0.0082	16
*PCNA*	0.0656	32	*CXCL2*	0.0060	11
*NRG1*	0.0575	19	*FOSL1*	0.0054	13
*SOX2*	0.0343	25	*TNFSF10*	0.0050	13
*PLAU*	0.0338	26	*PDCD4*	0.0042	13
*EPAS1*	0.0329	16	*EREG*	0.0032	10
*NCL*	0.0273	16	*AREG*	0.0026	11
*HSPB1*	0.0268	14	*H2AX*	0.0022	16

### Validation of hub genes in 3D air–liquid interface (ALI) system

To simulate in vivo inhalation exposure, we employed the 3D reconstructed human airway model SoluAirway and exposed it to aerosolized formaldehyde using a commercially available medical nebulizer. The concentration of formaldehyde exposure was set by tenfold serial dilutions (14.7 mg/mL (≈ 500 mM) to 50 μM), based on a previous study^21^. The MTT assay result showed that the tissue viability was > 90% at concentrations up to 5 mM and dropped significantly at concentrations above 50 mM (Supplementary Fig. S1). Among the sub-cytotoxic concentrations (> 90% viability), 0.5 mM (500 μM) was determined for qRT-PCR, considering that the 15-ppm data of the GEO dataset used is almost equivalent to 500 μM. The expression levels of the six hub genes were upregulated in the DEG results of all exposure periods and were validated using qRT-PCR. The mRNA expression of these genes examined in the 3D ALI system tended to be upregulated except *HMOX1* (Fig. 5b).

## Discussion

Formaldehyde is widely used in various fields and poses a high toxicity concern. In the early 1980s, the carcinogenicity of 5.6 and 14.3 ppm formaldehyde was confirmed in rats and mice for 2 years of exposure^23^. These concentrations are very high and unlikely to be encountered in daily life^24^. Therefore, our results using 15-ppm exposure data reflect high-exposure scenarios, such as chemical accidents and chronic occupational exposure to formaldehyde. Inhaled formaldehyde mainly affects the upper respiratory system, but a certain amount can be deposited directly into the lower respiratory system during oronasal breathing^2^. In animal, epidemiologic, and pulmonary function test studies, inhaled formaldehyde caused respiratory symptoms, with asthma identified as one of the most common diseases to arise due to exposure in indoor and occupational environments^25–29^. However, detailed knowledge of the mechanisms of respiratory disease onset associated with genetic changes in response to formaldehyde exposure is still lacking. This study explored integrated transcriptomic changes resulting from formaldehyde inhalation exposure.

Utilizing public genomic data, we acquired significant genetic profiles of formaldehyde exposure. We identified hub genes related to respiratory effects through network analyses and qRT-PCR validation via the 3D ALI aerosol exposure system (Figs. 4, 5). AREG, a ligand of epidermal growth factor receptor (EGFR, ErbB1), was the most strongly expressed, with a fold change > 20 in all exposure groups, and was enriched in ErbB Signaling. AREG activates cell signaling pathways, including Ras/Raf/MEK/ERK and PI3K/AKT, and cellular events, such as cell proliferation and apoptosis^30^. Elevated AREG expression is associated with airway inflammatory diseases, such as chronic obstructive pulmonary disease (COPD), asthma^31,32^, and several types of cancer^33^. HMOX1 is the major downstream antioxidant protein of the NRF2-mediated Oxidative Stress Response pathway and contributes to inhibiting Ferroptosis Signaling, an iron-dependent cell death accompanied by the accumulation of lipid reactive oxygen species^34,35^. HMOX1 is generally known to defend against oxidative and inflammatory damages in diverse diseases, and there are results of overexpressed HMOX1 in some respiratory patients^36–39^. However, the qRT-PCR result did not match the expression tendency of the microarray DEG results. This may be due to inherent differences in the tissues used in the bioassays as well as the exposure environment^40^. PTGS2 is not expressed in most tissues in the normal state, but its expression is induced by diverse stimuli, including cytokines, such as epidermal growth factor, interleukin-1, and tumor necrosis factor, and during an inflammatory response^41^. PTGS2 expression and cytokines were increased in the respiratory epithelium and alveolar macrophages of asthmatics^42,43^. Persistent increased *PTGS2* expression is thought to be induced by the inflammatory response after formaldehyde exposure^44,45^. PLAUR has been identified as an asthma, pneumonia, and COPD susceptibility gene^46,47^. PLAUR expression was elevated in the bronchial biopsy of asthmatics and is required for epithelial wound repair^48^. TIMP1 mainly functions as an important endogenous inhibitor in regulating matrix metalloproteinases. An imbalance of the MMP9/TIMP ratio is associated with the pathogenesis of asthma and lung diseases^49^. Sputum of asthmatics showed increased mRNA expression of *TIMP1*^50^. CXCL2 regulates normal and asthmatic airway smooth muscle cell migration^51^. During pulmonary inflammation, the mRNA expression of *Cxcl2* was increased in rats^52^. Taken together, selected hub genes could be important markers for respiratory diseases caused by inhaled toxicants. Furthermore, people with defects in these genes will be more vulnerable to formaldehyde exposure even if they are not exposed to high concentrations^53^.

Additionally, we compared our common DEGs with in vivo (human) and in vitro formaldehyde exposure data (GSE27263: nasal biopsy of volunteers exposed to formaldehyde up to 0.7 ppm for 4 h/day over 5 days; GSE21477: nasal epithelial cells exposed to 200 μM formaldehyde for 4 h). Interestingly, *NRARP* was identified as the only gene that showed increased expression in all formaldehyde-exposed data used, and 9 and 39 common genes were identified in human in vivo and in vitro data, respectively. Apart from the network analysis results, the up-regulation of the *NRARP* gene was also validated (Supplementary Fig. S2). Although the functions of NRARP and its association with formaldehyde have not been fully understood, it interacts with Notch and WNT signaling in angiogenesis^54^ and presents dual pro- or antitumor activity^55^. The *Notch1* gene positively regulates *Nrarp*^56^ and is upregulated in the long-term exposure (5 days to 13 weeks) DEG results. NRARP stimulates cell proliferation by negatively modulating p21/Rb-dependent cell cycle arrest^57^. Although the significance between the *NRARP* gene and respiratory diseases was not analyzed in the network analyses of our study, respiratory effects and cell cycle regulation-related carcinogenic effects of *NRARP* would be valuable in future formaldehyde studies.

Traditionally, inhalation toxicity studies are based on a strict animal inhalation test that is costly, time-consuming, technically challenging, and unethical. Many in vitro studies have relied on two-dimensional (2D) monocultures in submerged conditions that cannot reflect the real in vivo cellular environments and exposure conditions. Efforts to reduce the gap between animal testing and 2D cell culture models and reflect in vivo physiological conditions have led to advanced in vitro studies, such as 3D cell culture and microfluidics/microengineering technologies^58^. Additionally, studies incorporating a sophisticated aerosol exposure system (e.g., VITROCELL) and 3D reconstructed airway tissue to reflect actual exposure are considered ideal in studying inhalation toxicity^59^. For this reason, we employed a 3D reconstructed human airway model and exposed it to aerosolized formaldehyde to investigate the toxic effects of inhaled formaldehyde. Although consideration of interspecies differences and further studies on the reliability of 3D in vitro data will be needed, our integrated approach suggests an applicable analytic pipeline for predicting the toxic effects of inhaled toxicants.

This study performed a toxicogenomic in silico analysis based on in vivo data and validated the result in the 3D ALI system. However, our approach should be viewed as a prioritization method for further toxicity testing due to the limitations of data mining. For instance, the quality of data mining analysis is dependent on the databases of the software used^60^. Moreover, there could be false-positive results and missing interactions due to potential bias toward well-studied interactions^40,61^. Finally, further studies are needed to identify all possible molecular changes by formaldehyde exposure, considering other types of omics data and other factors, such as detailed dose–response relationships, duration, and sensitivity of the exposed individuals^62–64^.

## Conclusion

In summary, through a series of bioinformatic analyses of DEGs derived from public in vivo studies, hub genes related to respiratory diseases associated with formaldehyde exposure were identified. Several studies have evaluated lung damages caused by formaldehyde inhalation in rats^65,66^. Formaldehyde toxicity is considered to affect the entire respiratory system, not just the upper respiratory system. Considering these points, the significance of our toxicogenomic approach is that it can identify the respiratory effects of inhaled toxicants, serve as an early predictive alarm before serious lung diseases occur, and provide the possibility for identified genes to be used as biomarkers for clinical diagnosis and therapy. In addition, our results will contribute to improving the understanding of the toxic mechanism of formaldehyde, such as the construction of an adverse outcome pathway: suggestion of putative key events or adverse outcomes at diverse molecular levels, based on canonical pathways, cell processes, and diseases; inference of causal interaction; predictive assessment of carcinogenicity^67,68^.

## Supplementary Information

Supplementary Information.

## Data Availability

The datasets used during the current study are available in the NCBI Gene Expression Omnibus (accession number: GSE7002, GSE23179, GSE27263, and GSE21477). The other data generated or analyzed during the current study are available from the corresponding author upon reasonable request.

## References

[1] International Agency for Research on Cancer (2012). Chemical agents and related occupations. IARC Monogr. Eval. Carcinog. Risks Hum..

[2] National Toxicology Program. Final report on carcinogens background document for formaldehyde. *Rep. Carcinog. Backgr. Doc*. i-512 (2010). 20737003

[3] Costa S (2008). Genotoxic damage in pathology anatomy laboratory workers exposed to formaldehyde. Toxicology.

[4] Swenberg JA (2013). Formaldehyde carcinogenicity research: 30 years and counting for mode of action, epidemiology, and cancer risk assessment. Toxicol. Pathol..

[5] Nandan A, Siddiqui NA, Singh C, Aeri A (2021). Occupational and environmental impacts of indoor air pollutant for different occupancy: A review. Toxicol. Environ. Health Sci..

[6] Stewart PA, Cubit D, Blair A (1987). Formaldehyde levels in seven industries. Appl. Ind. Hygiene.

[7] Tang X (2009). Formaldehyde in China: production, consumption, exposure levels, and health effects. Environ. Int..

[8] Rim K-T (2021). Exposure of chemical mixtures at work and their application to the prevention of occupational disease. Toxicol. Environ. Health Sci..

[9] Clough E, Barrett T (2016). The gene expression omnibus database. Methods Mol. Biol..

[10] Barabasi AL, Oltvai ZN (2004). Network biology: understanding the cell's functional organization. Nat. Rev. Genet..

[11] Managbanag JR (2008). Shortest-path network analysis is a useful approach toward identifying genetic determinants of longevity. PLoS ONE.

[12] Andersen ME, Clewell HJ, Bermudez E, Willson GA, Thomas RS (2008). Genomic signatures and dose-dependent transitions in nasal epithelial responses to inhaled formaldehyde in the rat. Toxicol. Sci..

[13] Andersen ME (2010). Formaldehyde: Integrating dosimetry, cytotoxicity, and genomics to understand dose-dependent transitions for an endogenous compound. Toxicol. Sci..

[14] Irizarry RA (2003). Exploration, normalization, and summaries of high density oligonucleotide array probe level data. Biostatistics.

[15] Leek JT, Johnson WE, Parker HS, Jaffe AE, Storey JD (2012). The sva package for removing batch effects and other unwanted variation in high-throughput experiments. Bioinformatics.

[16] Ritchie ME (2015). limma powers differential expression analyses for RNA-sequencing and microarray studies. Nucleic Acids Res..

[17] Kramer A, Green J, Pollard J, Tugendreich S (2014). Causal analysis approaches in ingenuity pathway analysis. Bioinformatics.

[18] Nikitin A, Egorov S, Daraselia N, Mazo I (2003). Pathway studio–the analysis and navigation of molecular networks. Bioinformatics.

[19] Kang DS (2021). Formaldehyde exposure and leukemia risk: a comprehensive review and network-based toxicogenomic approach. Genes Environ..

[20] Assenov Y, Ramirez F, Schelhorn SE, Lengauer T, Albrecht M (2008). Computing topological parameters of biological networks. Bioinformatics.

[21] Lee, N. *et al.* Local toxicity of biocides after direct and aerosol exposure on the human skin epidermis and airway tissue models. *Toxics* **9**. 10.3390/toxics9020029 (2021). 10.3390/toxics9020029 PMC7913294 33546295

[22] Livak KJ, Schmittgen TD (2001). Analysis of relative gene expression data using real-time quantitative PCR and the 2(-Delta Delta C(T)) Method. Methods.

[23] Kerns WD, Pavkov KL, Donofrio DJ, Gralla EJ, Swenberg JA (1983). Carcinogenicity of formaldehyde in rats and mice after long-term inhalation exposure. Cancer Res..

[24] World Health Organization. *WHO Guidelines for Indoor Air Quality: Selected Pollutants*. WHO Guidelines Approved by the Guidelines Review Committee. p. 103–56. ISBN 9789289002134 (2010). 23741784

[25] Schachter EN, Witek TJ, Tosun T, Leaderer BP, Beck GJ (1986). A study of respiratory effects from exposure to 2 ppm formaldehyde in healthy subjects. Arch. Environ. Health.

[26] Malaka T, Kodama AM (1990). Respiratory health of plywood workers occupationally exposed to formaldehyde. Arch. Environ. Health.

[27] Lino dos Santos Franco, A. *et al.* Pulmonary neutrophil recruitment and bronchial reactivity in formaldehyde-exposed rats are modulated by mast cells and differentially by neuropeptides and nitric oxide. *Toxicol. Appl. Pharmacol.* **214**, 35–42. 10.1016/j.taap.2005.11.014 (2006). 10.1016/j.taap.2005.11.014 16427670

[28] Uthiravelu P, Saravanan A, Kumar CK, Vaithiyanandane V (2015). Pulmonary function test in formalin exposed and nonexposed subjects: A comparative study. J. Pharm. Bioallied Sci..

[29] Yu L (2020). Association between indoor formaldehyde exposure and asthma: A systematic review and meta-analysis of observational studies. Indoor Air.

[30] Berasain C, Avila MA (2014). Amphiregulin. Semin. Cell Dev. Biol..

[31] de Boer WI (2006). Expression of epidermal growth factors and their receptors in the bronchial epithelium of subjects with chronic obstructive pulmonary disease. Am. J. Clin. Pathol..

[32] Ogata-Suetsugu S (2017). Amphiregulin suppresses epithelial cell apoptosis in lipopolysaccharide-induced lung injury in mice. Biochem. Biophys. Res. Commun..

[33] Busser B, Sancey L, Brambilla E, Coll JL, Hurbin A (1816). The multiple roles of amphiregulin in human cancer. Biochim. Biophys. Acta.

[34] Loboda A, Damulewicz M, Pyza E, Jozkowicz A, Dulak J (2016). Role of Nrf2/HO-1 system in development, oxidative stress response and diseases: An evolutionarily conserved mechanism. Cell. Mol. Life Sci..

[35] Li J (2020). Ferroptosis: past, present and future. Cell Death Dis..

[36] Horvath I (1998). Raised levels of exhaled carbon monoxide are associated with an increased expression of heme oxygenase-1 in airway macrophages in asthma: A new marker of oxidative stress. Thorax.

[37] Harju T, Soini Y, Paakko R, Kinnula VL (2002). Up-regulation of heme oxygenase-I in alveolar macrophages of newly diagnosed asthmatics. Respir. Med..

[38] Mumby S (2004). Lung heme oxygenase-1 is elevated in acute respiratory distress syndrome. Crit. Care Med..

[39] Fredenburgh LE, Perrella MA, Mitsialis SA (2007). The role of heme oxygenase-1 in pulmonary disease. Am. J. Respir. Cell Mol. Biol..

[40] BARALIć K (2022). Potential genomic biomarkers of obesity and its comorbidities for phthalates and bisphenol A mixture: In silico toxicogenomic approach. Biocell.

[41] Williams CS, Mann M, DuBois RN (1999). The role of cyclooxygenases in inflammation, cancer, and development. Oncogene.

[42] Profita M (2003). Increased prostaglandin E2 concentrations and cyclooxygenase-2 expression in asthmatic subjects with sputum eosinophilia. J. Allergy Clin. Immunol..

[43] Peebles RS (2019). Prostaglandins in asthma and allergic diseases. Pharmacol. Ther..

[44] Lee S-H (2020). Anti-inflammatory effect of Rosa laevigata extract on in vitro and in vivo model of allergic asthma via the suppression of IgE and related cytokines. Mol. Cell. Toxicol..

[45] Zhang W (2021). Dapk1 promoted inflammation of infantile pneumonia by p38MAPK/NF-κB signaling pathway. Mol. Cell. Toxicol..

[46] Portelli MA (2014). Genome-wide protein QTL mapping identifies human plasma kallikrein as a post-translational regulator of serum uPAR levels. FASEB J..

[47] Wrotek A, Jackowska T (2015). The role of the soluble urokinase plasminogen activator (suPAR) in children with pneumonia. Respir. Physiol. Neurobiol..

[48] Stewart CE, Nijmeh HS, Brightling CE, Sayers I (2012). uPAR regulates bronchial epithelial repair in vitro and is elevated in asthmatic epithelium. Thorax.

[49] Chaudhuri R (2014). Low sputum MMP-9/TIMP ratio is associated with airway narrowing in smokers with asthma. Eur. Respir. J..

[50] Cataldo DD (2004). Matrix metalloproteinases and tissue inhibitors of matrix metalloproteinases mRNA transcripts in the bronchial secretions of asthmatics. Lab. Invest..

[51] Al-Alwan LA (2013). Differential roles of CXCL2 and CXCL3 and their receptors in regulating normal and asthmatic airway smooth muscle cell migration. J. Immunol..

[52] Huang S, Paulauskis JD, Godleski JJ, Kobzik L (1992). Expression of macrophage inflammatory protein-2 and KC mRNA in pulmonary inflammation. Am. J. Pathol..

[53] Miller MC, Mohrenweiser HW, Bell DA (2001). Genetic variability in susceptibility and response to toxicants. Toxicol. Lett..

[54] Phng LK (2009). Nrarp coordinates endothelial Notch and Wnt signaling to control vessel density in angiogenesis. Dev. Cell.

[55] Pinto I (2020). NRARP displays either pro- or anti-tumoral roles in T-cell acute lymphoblastic leukemia depending on Notch and Wnt signaling. Oncogene.

[56] Imaoka T (2014). Overexpression of NOTCH-regulated ankyrin repeat protein is associated with breast cancer cell proliferation. Anticancer Res..

[57] Korn C, Augustin HG (2015). Mechanisms of vessel pruning and regression. Dev. Cell.

[58] Huh D, Hamilton GA, Ingber DE (2011). From 3D cell culture to organs-on-chips. Trends Cell Biol..

[59] Neilson L (2015). Development of an in vitro cytotoxicity model for aerosol exposure using 3D reconstructed human airway tissue; application for assessment of e-cigarette aerosol. Toxicol. In Vitro.

[60] Živančević K (2021). Elucidating the influence of environmentally relevant toxic metal mixture on molecular mechanisms involved in the development of neurodegenerative diseases: In silico toxicogenomic data-mining. Environ. Res..

[61] Harris SM (2020). Identification of environmental chemicals targeting miscarriage genes and pathways using the comparative toxicogenomics database. Environ. Res..

[62] Suvorov A (2021). Unbiased approach for the identification of molecular mechanisms sensitive to chemical exposures. Chemosphere.

[63] Baralić K (2021). Probiotic reduced the impact of phthalates and bisphenol A mixture on type 2 diabetes mellitus development: Merging bioinformatics with in vivo analysis. Food Chem. Toxicol..

[64] Bozic D (2022). Predicting sulforaphane-induced adverse effects in colon cancer patients via in silico investigation. Biomed. Pharmacother..

[65] Murta GL (2016). Oxidative effects on lung inflammatory response in rats exposed to different concentrations of formaldehyde. Environ. Pollut..

[66] Liu QP (2018). Formaldehyde inhalation triggers autophagy in rat lung tissues. Toxicol. Ind. Health.

[67] Kim HS (2020). Suggestions for applications of toxicogenomic approaches in the adverse outcome pathway of 2,4-dinitrotoluene. Toxicol. Environ. Health Sci..

[68] Rim K-T (2021). Application of the adverse outcome pathway framework to predict the toxicity of chemicals in the semiconductor manufacturing industry. Mol. Cell. Toxicol..

