# Supplementary material for: Network-based integrated analysis for toxic effects of high-concentration formaldehyde inhalation exposure through the toxicogenomic approach
Source: Sci Rep. 2022 Apr 4;12:5645. doi: 10.1038/s41598-022-09673-0 (PMC8979994; doi:10.1038/s41598-022-09673-0)
Supplement: Supplementary file 1 — Supplementary Information. [file 41598_2022_9673_MOESM1_ESM.docx]

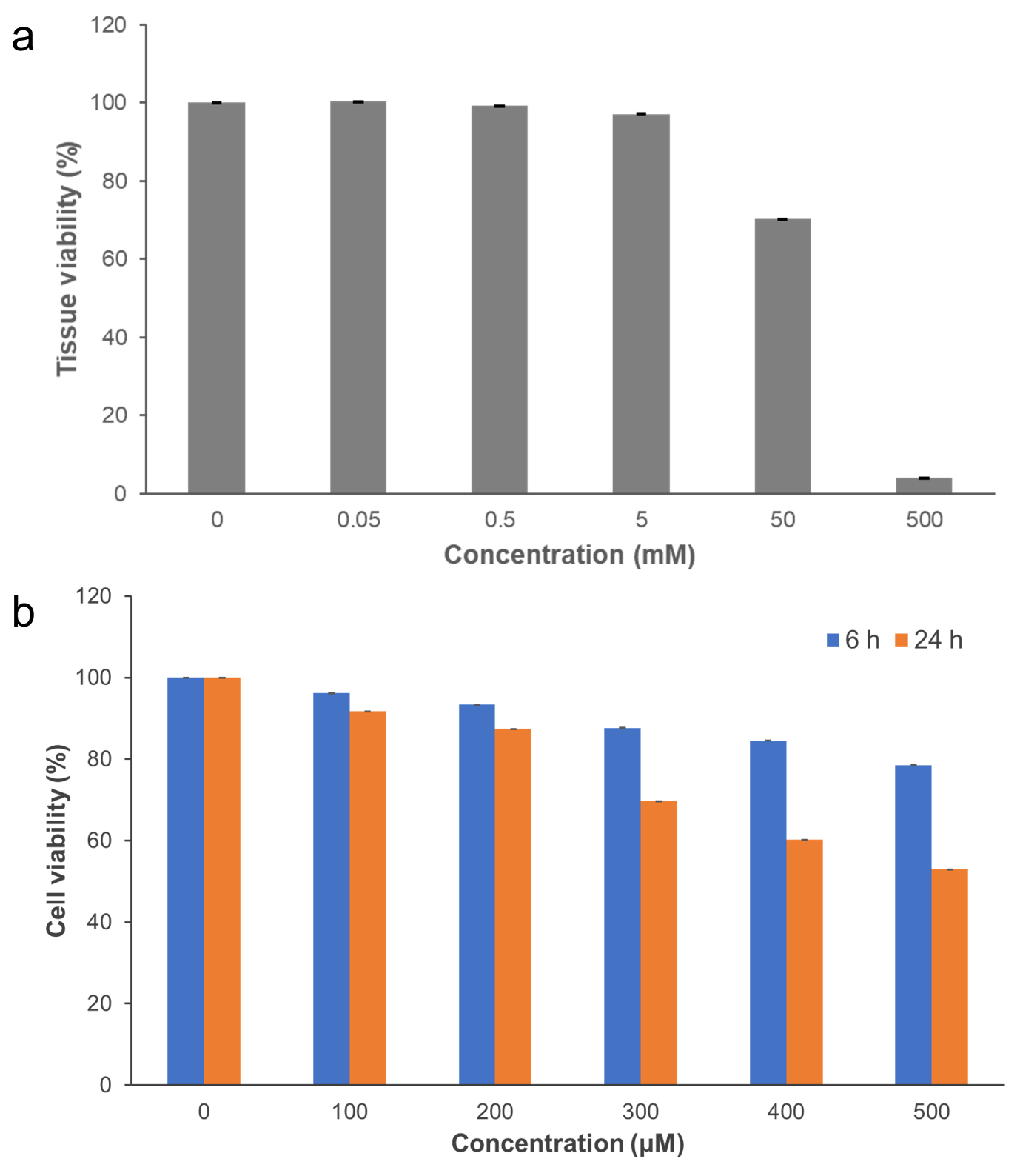


**Supplementary Figure S1.** Toxicity of formaldehyde. (**a**) MTT assay result in the 3D reconstructed human airway model (SoluAirway) exposed to aerosolized formaldehyde for 24 h. (**b**) MTT assay result in BEAS-2B cells. Prior to conducting MTT assay in the 3D exposure system, cytotoxicity of formaldehyde was pretested in cell line for 6 and 24 h. Error bars represent ± SEM.


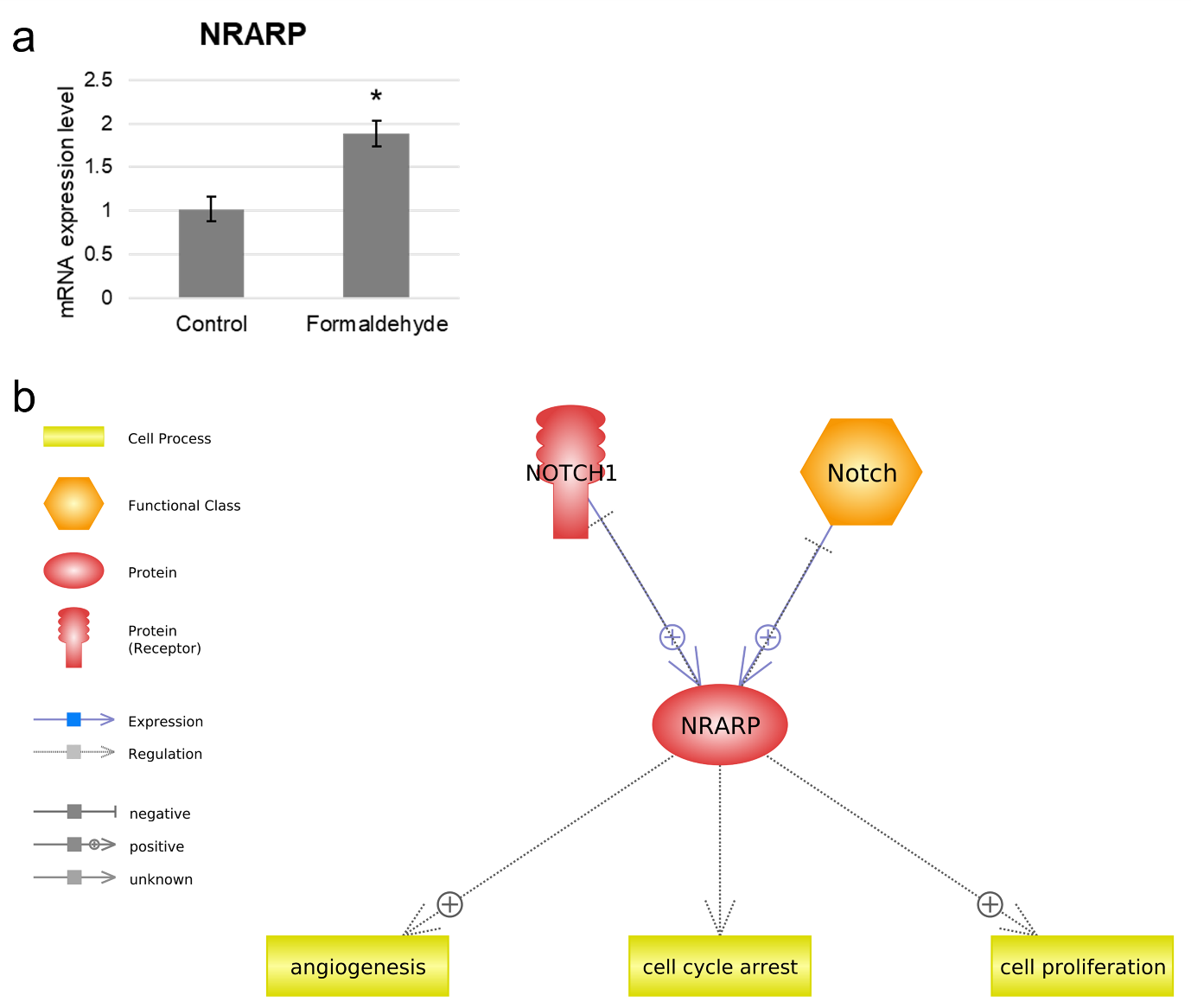


**Supplementary Figure S2.** *NRARP* gene expression and interactions. (**a**) Validation of the expression level of *NRARP* gene in SoluAirway. Single asterisk (*) indicates *p*-value < 0.05. (**b**) Biological interactions of *NRARP* gene using Pathway Studio (version 12.3).

**Supplementary Table S1.** Primer sequences for qRT-PCR.

| Gene | Forward/Reverse | Sequence (5′→3′) |
| --- | --- | --- |
| *AREG* | Forward | GGA GCC GAC TAT GAC TAC T |
|  | Reverse | CAC ACC GTT CAC CGA AAT A |
| *CXCL2* | Forward | TGG CAA ATC CAA CTG ACC |
|  | Reverse | ACA CAT TAG GCG CAA TCC |
| *HMOX1* | Forward | CCT CCC TGT ACC ACA TCT AT |
|  | Reverse | GGC TTT CTG GGC AAT CTT |
| *PLAUR* | Forward | ACA ACA ACG ACA CCT TCC |
|  | Reverse | AGT ACA GCA GGA GAC ATC A |
| *PTGS2* | Forward | GCC TGG TCT GAT GAT GTA TG |
|  | Reverse | GAA AGG TGT CAG GCA GAA G |
| *TIMP1* | Forward | TTG TTG CTG TGG CTG ATA G |
|  | Reverse | ACG CTG GTA TAA GGT GGT |
| *GAPDH* | Forward | ATG GGG AAG GTG AAG GTC G |
|  | Reverse | GGG GTC ATT GAT GGC AAC AA |
| *NRARP* | Forward | TCT TGT GCT AAT GGC TGA G |
|  | Reverse | AAA GTG ACG CAG GAG AAC |
